# McKittrick–Wheelock Syndrome Caused by Giant Tubulovillous Adenomas With Malignant Transformation: A Case Report

**DOI:** 10.1155/crgm/1802324

**Published:** 2026-06-23

**Authors:** Marko Kalezic, Emilija Krsmanovic, Srdjan Marinkovic, Miljan Milanovic, Djordje Miladinovic, Branka Filipovic, Predrag Savic, Dusan Popovic

**Affiliations:** ^1^ Department of Surgery, Clinic for Surgery, Clinical Hospital Center “Dr. Dragisa Misovic-Dedinje”, Belgrade, Serbia; ^2^ Department of Gastroenterology and Hepatology, Clinic for Internal Medicine, Clinical Hospital Center “Dr. Dragisa Misovic-Dedinje”, Belgrade, Serbia; ^3^ Department of Surgery With Anesthesiology, Faculty of Medicine, University of Belgrade, Belgrade, Serbia, bg.ac.rs; ^4^ Department of Internal Medicine, Faculty of Medicine, University of Belgrade, Belgrade, Serbia, bg.ac.rs

**Keywords:** adenocarcinoma, adenoma, case report, colonoscopy, diarrhea

## Abstract

Giant villous adenomas of the colon are a rare cause of chronic secretory diarrhea. These lesions can produce excessive mucus, fluid, and electrolyte loss, leading to life‐threatening hypokalemia, hyponatremia, and acute kidney injury. Chronic secretory diarrhea with electrolyte imbalance and possible acute kidney injury caused by secretory giant tubulovillous adenomas constitutes McKittrick–Wheelock syndrome, a rare condition described in 257 patients worldwide. We report two cases of McKittrick–Wheelock syndrome caused by giant tubulovillous adenomas of the rectum and rectosigmoid junction with malignant transformation. The first case, a 72‐year‐old female, presented with profound electrolyte depletion and acute renal failure, managed in the intensive care unit with supportive therapy. The second case, a 73‐year‐old male, had chronic diarrheal syndrome and marked electrolyte depletion due to three giant colonic tubulovillous adenomas with malignant transformation. After rehydration and electrolyte replacement, both patients underwent surgical resection with low anterior rectal resection and protective loop ileostomy. The male patient required reoperation with total proctocolectomy due to coloanal anastomotic dehiscence. Postoperative courses were uneventful, and both patients were discharged with normalized laboratory values and scheduled for regular follow‐up. McKittrick–Wheelock syndrome should be suspected in patients with chronic diarrhea and electrolyte disturbances. Prompt endoscopic evaluation allows accurate diagnosis, and timely fluid and electrolyte replacement combined with surgical resection of the secretory lesion is essential for effective treatment and prevention of serious complications.

## 1. Introduction

Chronic diarrhea is a common problem, with a reported prevalence of up to 5% in the adult population, depending on the population studied and the definition used [1]. It is defined as a change in stool consistency, increased bowel movement frequency, urgency, or incontinence lasting longer than 4 weeks [1]. Several mechanisms can cause chronic diarrhea; of these, secretory diarrhea persists despite fasting and is independent of food intake [1–3]. The most common causes include toxins from infectious agents (such as *Staphylococcus*, *E. coli*, and Vibrio cholerae) and hormone‐mediated hypersecretion (such as VIP, gastrin, and calcitonin) [3].

Polypoid colorectal tumors, predominantly villous adenomas, are a rare cause of chronic secretory diarrhea [4]. These tumors can secrete large amounts of fluid and mucus, potentially leading to electrolyte disturbances (hyponatremia, hypokalemia, and hypochloremia) and acute kidney injury. The clinical triad of secretory diarrhea, refractory electrolyte imbalance, and acute kidney injury due to a giant villous colon polyp was first described by Garis et al. in 1941 and later detailed by McKittrick and Wheelock in 1954, leading to the eponymous syndrome [4]. McKittrick–Wheelock syndrome is uncommon. The largest literature review identified 257 cases worldwide, though the true incidence may be higher, as the characteristic clinical and laboratory features are not always recognized as a single syndrome [5].

We report two patients presenting with McKittrick–Wheelock syndrome secondary to giant rectal villous adenomas.

## 2. Cases Presentations

### 2.1. Case # 1

A 72‐year‐old woman was admitted to the intensive care unit with general weakness, recurrent episodes of impaired consciousness, and two months of persistent diarrhea. Initial laboratory tests revealed severe hypokalemia, hyponatremia, metabolic acidosis, and acute renal failure.

The patient had previously undergone hysterectomy for uterine cancer, followed by radiotherapy and chemotherapy several years earlier. She had no other comorbidities and was not taking any regular medications. Family history of colorectal carcinoma was negative.

On admission, the patient was conscious, bradypsychic, afebrile, and hemodynamically stable (blood pressure 115/80 mmHg and heart rate 90/min). Physical examination was unremarkable. The abdomen was soft and nontender on both superficial and deep palpation. The liver and spleen were not palpable, without signs of ascites. Digital rectal examination revealed a sessile soft‐tissue mass approximately 6 cm from the anal verge, with copious mucus observed on the examining glove.

Laboratory findings revealed severe electrolyte disturbances, including hypokalemia (K 2.6 mmol/L), hyponatremia (Na 116 mmol/L), and hypochloremia (Cl 62 mmol/L), as well as low bicarbonate levels (HCO_3_^-^ 18 mmol/L) and acute renal failure (creatinine 443 μmol/L, urea 55.7 mmol/L, eGFR 9 mL/min/1.73 m^2^), while other biochemical parameters were within the reference range. The complete blood count revealed leukocytosis with neutrophil predominance (WBC 16.4 × 10^9^/L, neutrophils 14.68 × 10^9^/L) and elevated hemoglobin (Hb 190 g/L). Other hematological parameters were within normal limits. Stool cultures were negative for pathogenic bacteria, and C. difficile testing was also negative.

Abdominal ultrasonography demonstrated gaseous distension of the bowel and a suspected gallbladder calculus. A plain abdominal radiograph showed no pneumoperitoneum or air–fluid levels, and chest radiography revealed no acute abnormalities.

Colonoscopy performed during the same hospitalization revealed a giant sessile villous polyp of the rectosigmoid colon, extending from 3–4 cm to 20 cm from the anal verge, involving nearly the entire colonic circumference, and covered with copious mucus (Figure 1). Biopsy specimens revealed a low‐grade tubulovillous adenoma on histopathological analysis (Figure 2).

**FIGURE 1:**
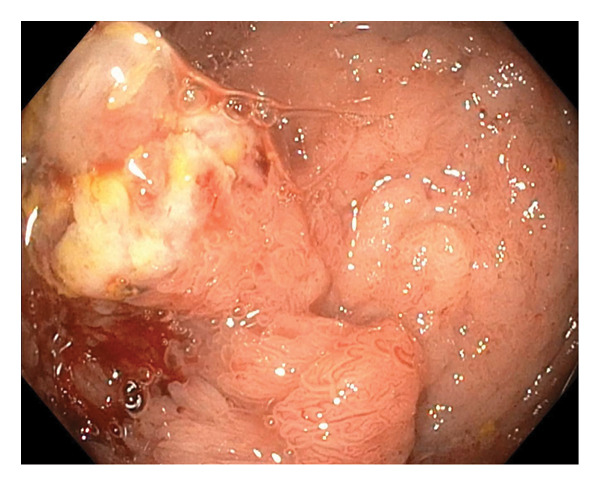
Case #1. Colonoscopy showing a giant sessile villous polyp in the rectosigmoid colon, involving nearly the entire colonic circumference and covered with copious mucus.

**FIGURE 2:**
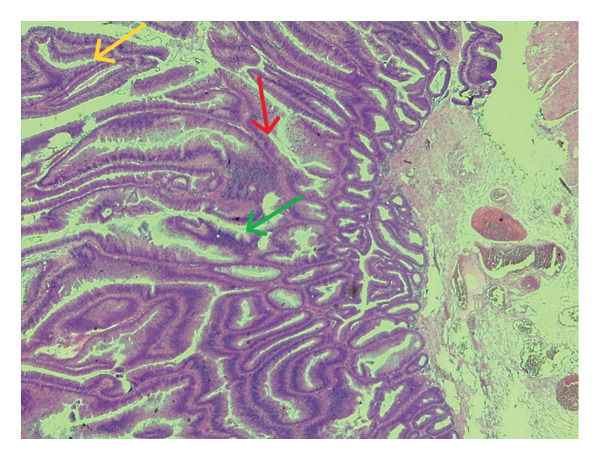
Case #1. Histopathological findings demonstrating a tubulovillous adenoma with low‐grade dysplasia. The lesion demonstrates a predominantly villous architecture with elongated dysplastic glands lined by pseudostratified columnar epithelium. Legend: yellow arrow–villous component; red arrow–low‐grade dysplasia; green arrow–tubular component (H&E stain, × 2.5).

Abdominal and pelvic CT confirmed an intraluminal tumor involving the rectum and rectosigmoid junction, without evidence of invasion into surrounding tissues or locoregional or distant metastases (Figure 3). Incidental cholelithiasis was also noted.

**FIGURE 3:**
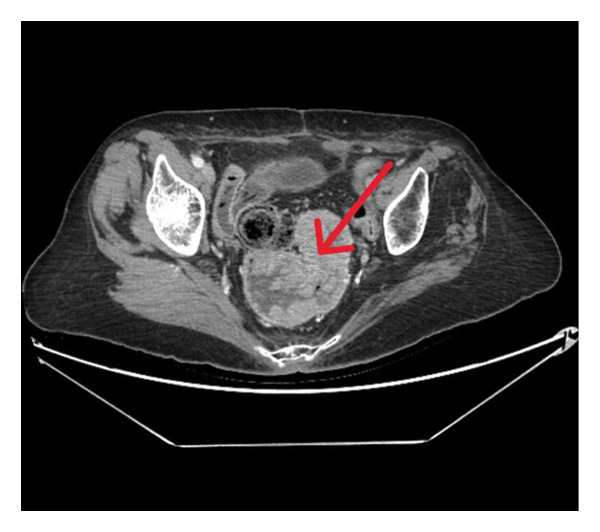
Case #1. Abdominal and pelvic CT demonstrating an intraluminal tumor (red arrow) involving the rectum and rectosigmoid junction.

Specialist consultations in nephrology, cardiology, and endocrinology were performed to rule out alternative causes of severe renal failure beyond the diarrheal syndrome. She received nonsurgical management, including aggressive intravenous fluid therapy and correction of electrolyte imbalances, resulting in rapid improvement of clinical condition and laboratory parameters. The patient was discharged after 12 days of hospitalization, with normalization of electrolyte levels (K 3.5 mmol/L, Na 138 mmol/L, and HCO_3_^-^ 29 mmol/L) and renal function (urea 2.3 mmol/L, creatinine 58 μmol/L, and eGFR 94.1 mL/min/1.73 m^2^).

The patient was readmitted to the Department of Surgery 7 days after discharge for elective surgical resection. On admission, laboratory tests again demonstrated severe electrolyte disturbances (K 3.0 mmol/L, Na 126 mmol/L, Cl 88 mmol/L) and mild renal impairment (creatinine 92 μmol/L, urea 5.9 mmol/L, eGFR 55.3 mL/min/1.73 m^2^). The completed blood count showed normal leukocyte and hemoglobin levels with neutrophilia and mild reactive thrombocytosis, and CRP was elevated (47.8 mg/L), likely secondary to a urinary tract infection.

Following preoperative preparation, the patient underwent open surgery on day five. Intraoperatively, a large tumor from the rectosigmoid junction was found occupying the pelvis. An ultra‐low anterior resection with colorectal end‐to‐end anastomosis and protective loop ileostomy was performed, along with an appendectomy and cholecystectomy due to gallbladder lithiasis (Figure 4).

**FIGURE 4:**
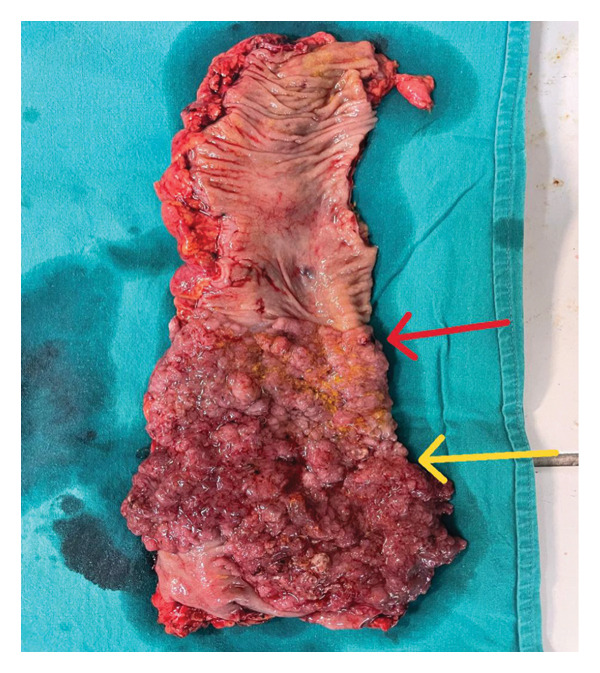
Case #1. Intraoperative specimen showing a large tumor (yellow arrow) originating from the rectosigmoid junction (red arrow) and occupying the pelvis.

Histopathology revealed a well‐differentiated invasive colonic adenocarcinoma grade I (pTNM: T2N0 [0/4] Mx; Dukes A; Astler–Coller B1) arising from a tubulovillous adenoma with both low‐ and high‐grade dysplasia, infiltrating the muscularis propria without evidence of metastasis (Figure 5).

**FIGURE 5:**
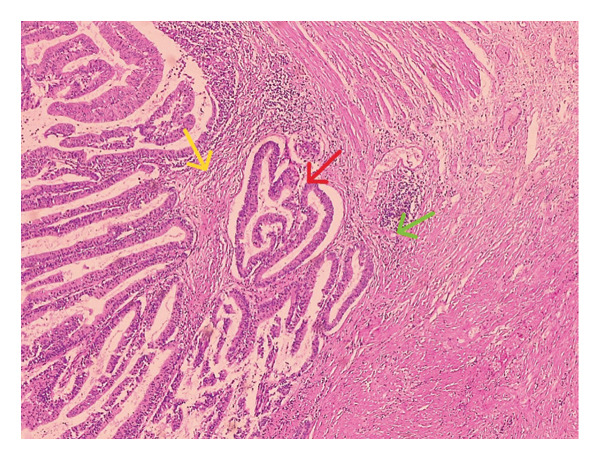
Case #1. Histopathological findings demonstrating grade 1 adenocarcinoma with infiltration of the muscularis propria. Legend: yellow arrow–adenoma‐to‐carcinoma transition area; red arrow–invasive adenocarcinoma gland; green arrow–muscularis propria infiltrated by adenocarcinoma (H&E stain, × 5).

The postoperative course was complicated by urinary bladder distension caused by catheter displacement; the catheter was repositioned. Electrolyte balance and renal function normalized by discharge, which occurred on postoperative day seven, with scheduled surgical and urological follow‐up.

At the first postoperative surgical follow‐up visit, the patient was asymptomatic. The ileostomy functioned properly, the wound was healing normally, and laboratory values were within the reference range.

### 2.2. Case #2

A 73‐year‐old man was admitted to our clinic for surgical resection of a rectal polyp. For the past 3 years, he had experienced frequent watery and mucoid stools alternating with formed or soft stools. He had not attended regular follow‐up visits. Several months prior to admission, his symptoms worsened, with more frequent mucus per rectum, nausea, and muscle cramps in the lower extremities. Colonoscopy revealed a large polypoid lesion of the rectum, which could not be resected endoscopically. Biopsy demonstrated a tubulovillous adenoma with low‐grade dysplasia.

His previous medical history included two surgeries for urothelial carcinoma of the urinary bladder, followed by adjuvant intravesical BCG therapy and radiotherapy, and secondary epilepsy due to head trauma 20 years prior to admission. Family history of colorectal carcinoma was negative.

On admission, the patient was conscious, oriented, afebrile, eupneic, acyanotic, anicteric, and hemodynamically stable (blood pressure 110/70 mmHg; heart rate 61/min). The abdomen was soft and nontender. Digital rectal examination revealed a soft, lobulated mass on the anterior rectal wall, 6 cm from the anocutaneous line, with traces of yellow stool and abundant mucus on the glove.

Laboratory analyses confirmed severe hypokalemia (K 2.8 mmol/L), hypobicarbonatemia (HCO_3_^-^ 20 mmol/L), and hyperchloremia (Cl 108 mmol/L). Sodium levels and renal function were normal. The completed blood count showed mild leukocytosis (10.5 × 10^9^/L), with neutrophilia (8.19 × 10^9^/L), normal hemoglobin, and platelets. CRP was normal. CA 19‐9 and D‐dimer were elevated (CA 19‐9 98.7 U/mL, D‐dimer 1.47 μg/mL).

Ultrasound examination of the abdomen was unremarkable.

Colonoscopy was performed up to 25 cm from the anocutaneous line. From 6 to 25 cm, a large polypoid lesion occupied approximately two‐thirds of the lumen, preventing further passage of the endoscope (Figure 6).

**FIGURE 6:**
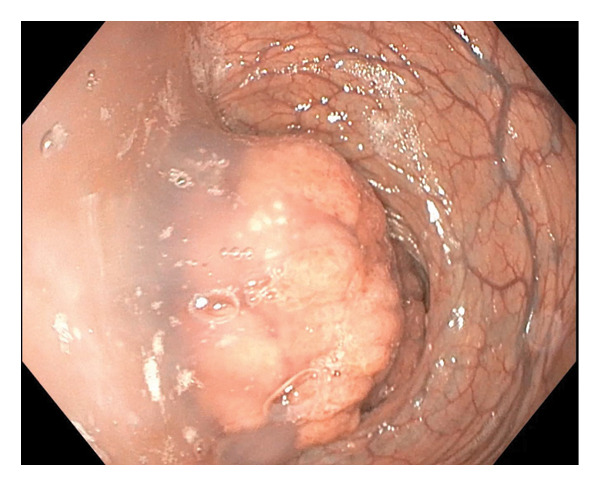
Case #2. Colonoscopy showing a large polypoid lesion occupying approximately two‐thirds of the lumen and preventing further passage of the endoscope, with abundant mucus.

CT of the abdomen and pelvis revealed a large rectal polypoid lesion with suspected sigmoid volvulus, a polypoid lesion at the hepatic flexure, and a stenotic lesion of the transverse colon suggestive of an infiltrative process (Figure 7). No metastatic lesions were detected. CT of the chest was normal. Pelvic MRI showed a lesion extending from 6 cm above the anocutaneous line to 9 cm proximally.

**FIGURE 7:**
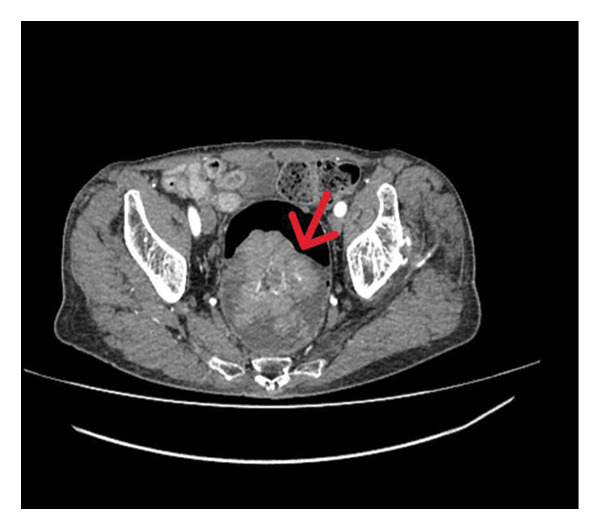
Case #2. Abdominal and pelvic CT revealing a large rectal polypoid lesion (red arrow) with suspected sigmoid volvulus.

After adequate preoperative preparation, the patient underwent open surgery on the fourth day of hospitalization. Intraoperatively, a large intraluminal tumor of the sigmoid colon invaginating into the rectum was found, along with lesions of the transverse colon (5–6 cm) and below the hepatic flexure (3 cm) (Figure 8). An extended right hemicolectomy, sigmoid resection, and low anterior resection (RRAI) with total mesorectal excision (TME) were performed, preserving the left colic vessels. Ileocolic side‐to‐side and coloanal end‐to‐end stapled anastomoses were created, with a protective loop ileostomy and surgical drain placement.

**FIGURE 8:**
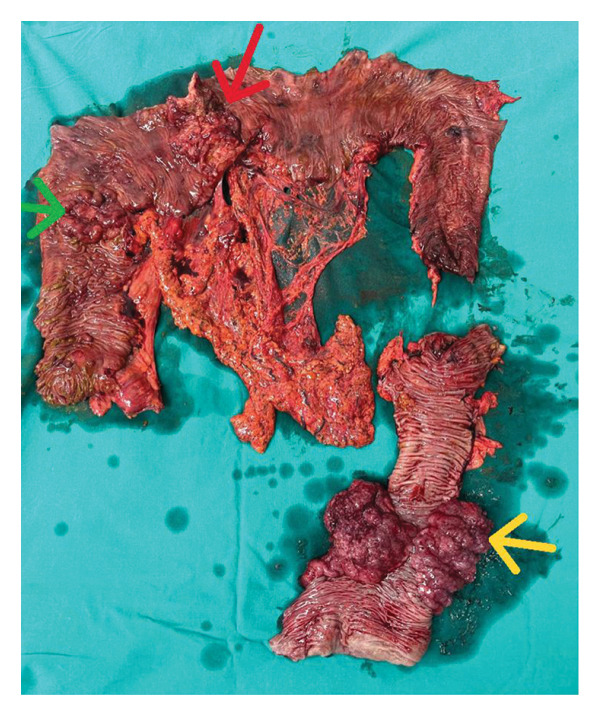
Case #2. Intraoperative specimen showing a large intraluminal tumor of the sigmoid colon (yellow arrow), along with additional lesions in the transverse colon (red arrow) and distal to the hepatic flexure (green arrow).

Final histopathology revealed a well‐differentiated adenocarcinoma of the rectosigmoid segment, grade 1 (pT2N0M0, Dukes A, Astler–Coller B1); a moderately differentiated adenocarcinoma of the ascending colon, grade 2 (pT1N0M0, Dukes A, Astler–Coller B1) (Figure 9); and a synchronous poorly differentiated adenocarcinoma of the transverse colon with a mucinous component (5%), grade 3 (pT3N0M0, Dukes B, Astler–Coller B1). The rectosigmoid and ascending colon tumors arose from malignant transformation of pre‐existing tubulovillous adenomas.

**FIGURE 9:**
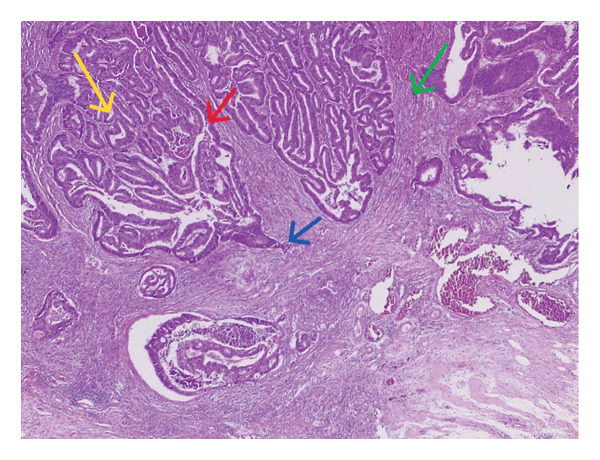
Case #2. Histopathological findings demonstrating adenocarcinoma arising in a tubulovillous adenoma with submucosal infiltration. Legend: yellow arrow–tubulovillous adenoma; red arrow–adenoma‐to‐carcinoma transition; green arrow–adenocarcinoma; blue arrow–submucosal infiltration (H&E stain, × 2.5).

Postoperatively, the patient remained hemodynamically stable, without systemic inflammation, and with unremarkable abdominal findings. Due to abdominal pain, follow‐up endoscopy revealed venous congestion of the remaining left colon and coloanal anastomosis. The patient was managed conservatively. By the end of hospitalization, the anastomosis and remaining colon were viable, the ileostomy was functional, oral intake was initiated, and laboratory parameters were normalized. The patient was discharged on hospital Day 15.

Seven days after discharge, the patient developed fatigue and mild periumbilical pain. Clinical and colonoscopic findings confirmed anastomotic dehiscence, ischemia of the remaining colon, and localized peritonitis. The patient was re‐admitted, and a total proctocolectomy with terminal ileostomy was performed. The postoperative course was uneventful, and he was discharged after 14 days with normal laboratory results and no subjective complaints. The case was reviewed by the Multidisciplinary Tumor Board, which decided on regular follow‐up without specific oncological treatment. In the later course, at the regular surgical follow‐up visits, the patient is subjectively well, with normal clinical and laboratory findings, and the terminal ileostomy remains functional.

## 3. Discussion

Approximately 5% of colorectal adenomas are villous; among these, around 3% of tumors larger than 3–4 cm display secretory activity capable of causing electrolyte depletion [5, 6]. Tumor dimensions in one study ranged from 7 to 18 cm [7].

In addition to their large size, excessive mucus production can be explained by histological findings showing that the epithelium is largely composed of mucus‐producing goblet cells, including atypical goblet cells secreting abnormal mucin [7, 8]. Additional pathophysiological mechanisms of hypersecretory activity in villous adenomas associated with McKittrick–Wheelock syndrome were investigated in an in vitro study by Jacobs et al. [9]. They demonstrated that cAMP‐dependent protein kinase activity in tumor tissue and adjacent mucosa was up to three times higher than in normal colonic mucosa, leading to increased production of PGE2 (approximately 3–6 times higher), which could be suppressed by indomethacin [9].

In McKittrick–Wheelock syndrome, tumors are typically located in the rectosigmoid colon, as in our patients. The larger and more distally located the lesion/tumor, the lower the ability of the surrounding normal mucosa to reabsorb electrolytes and water, increasing the risk of severe diarrheal syndrome. Clinical manifestations include secretory diarrhea, dehydration, electrolyte disturbances (hyponatremia and hypokalemia), and metabolic acidosis, presenting with headache, nausea, vomiting, muscle cramps, and in more severe cases, seizures and cardiac arrhythmias [6, 10]. In both our patients, in addition to secretory diarrhea, severe hypokalemia and metabolic acidosis were present.

Colorectal adenomas are common neoplasms with variable malignant potential. The risk of malignant transformation increases with adenoma size greater than 1 cm, distal location, and villous histologic features [11]. Tumors larger than 2 cm with a significant villous component have an estimated 50% risk of malignant transformation [12].

Recent reports indicate that large tubulovillous adenomas and adenocarcinomas can show hypersecretory behavior akin to large villous adenomas [5]. Both patients in our study had adenocarcinomas originating from pre‐existing adenomas, resulting in pronounced secretory activity and McKittrick–Wheelock syndrome. The male patient also had a synchronous mucinous adenocarcinoma, potentially contributing to his presentation.

Since secretory lesions cause the symptoms, lesion removal prevents McKittrick–Wheelock syndrome and malignant transformation, which is common in large villous tumors. Endoscopic resection is preferred, but for large lesions, surgical resection is often necessary and effective, as demonstrated in our two cases.

In selected cases, patients with a solitary, distal tumor; favorable histological features (e.g., low‐grade dysplasia and T1 carcinoma); and no need for RRAI may undergo minimally invasive procedures such as transanal mucosectomy in continuity and rectal resection with hand‐sewn coloanal anastomosis, achieving favorable outcomes [13].

According to recent reports, laparoscopic rectal resection with TME and proctosigmoidectomy is suitable for treating giant villous adenomas of the rectosigmoid colon associated with McKittrick–Wheelock syndrome [10, 14].

McKittrick–Wheelock syndrome may be life‐threatening if not treated due to severe dehydration, prerenal azotemia, and possible acute kidney injury. Fluid and electrolyte replacement is a key component of therapy and essential prior to curative surgery. Indomethacin can reduce rectal fluid loss by up to 50%, whereas somatostatin analogues may paradoxically increase it by around 25% [9, 14].

Because of the condition’s complexity, a multidisciplinary approach is often required [5, 15]. Our female patient underwent evaluation by a nephrologist, cardiologist, and endocrinologist to exclude other causes of severe renal failure unrelated to the diarrheal syndrome.

Given the absence of disease‐specific surveillance protocols for McKittrick–Wheelock syndrome, postoperative follow‐up should be guided by current colorectal cancer surveillance recommendations, particularly in patients with malignant transformation or synchronous neoplasms. Since malignant transformation of tubulovillous adenomas was identified in both of our patients, long‐term surveillance was planned according to current colorectal cancer follow‐up principles. According to European Society for Medical Oncology (ESMO) guidelines [16], follow‐up includes regular clinical and laboratory assessment with carcinoembryonic antigen (CEA) monitoring every 3–6 months during the first 3 years, as well as periodic computed tomography of the chest, abdomen, and pelvis when clinically indicated. Surveillance colonoscopy is generally recommended 1 year after curative resection and subsequently at 3–5‐year intervals. However, earlier endoscopic reassessment may be justified in selected high‐risk patients, particularly in cases with giant villous lesions, synchronous neoplasms, incomplete preoperative colonic evaluation, or postoperative complications. In our patients, individualized and more intensive follow‐up was considered appropriate because both had a history of additional primary malignancies (uterine carcinoma in the first patient and urothelial carcinoma in the second patient), potentially indicating an increased overall oncologic risk.

As this syndrome is uncommon and can resemble other conditions, prompt recognition requires a high index of suspicion. Colorectal adenoma should be considered when prerenal insufficiency, electrolyte imbalance, and chronic diarrhea coexist, and complete colonoscopic evaluation is advised.

## 4. Conclusion

McKittrick–Wheelock syndrome is a rare but potentially life‐threatening condition that should be considered in patients presenting with chronic secretory diarrhea, dehydration, and electrolyte imbalance. Early recognition, adequate fluid and electrolyte replacement, and timely surgical removal of the secretory colorectal lesion are essential for preventing serious complications. Complete colonoscopic evaluation is mandatory when this clinical triad is present, as large villous adenomas or synchronous colorectal neoplasms may be the underlying cause.

## Author Contributions

Marko Kalezic and Dusan Popovic designed the overall concept and outline of the manuscript; Marko Kalezic, Emilija Krsmanovic, Srdjan Marinkovic, Miljan Milanovic, Djordje Miladinovic, Branka Filipovic, Predrag Savic, and Dusan Popovic contributed to the acquisition, analysis, and interpretation of the data; Emilija Krsmanovic and Dusan Popovic drafted the article; Marko Kalezic, Emilija Krsmanovic, and Dusan Popovic made critical revisions related to important intellectual content of the manuscript.

## Funding

This work was supported by the Ministry of Science, Technological Development and Innovations, Republic of Serbia, No. 451‐03‐137/2025‐03/200110.

## Disclosure

All authors approved the final version of the article.

## Ethics Statement

This case report was conducted in accordance with institutional ethical standards and the principles of the Declaration of Helsinki. Written informed consent was obtained from the patients for publication of this case report and the accompanying images.

## Conflicts of Interest

The authors declare no conflicts of interest.

## Data Availability

The data that support the findings of this study are available from the corresponding author upon reasonable request.
